# Seizure Duration and Electroconvulsive Therapy in Major Depressive Disorder

**DOI:** 10.1001/jamanetworkopen.2024.22738

**Published:** 2024-07-25

**Authors:** Cecilia Gillving, Carl Johan Ekman, Åsa Hammar, Mikael Landén, Johan Lundberg, Pouya Movahed Rad, Pia Nordanskog, Lars von Knorring, Axel Nordenskjöld

**Affiliations:** 1 Faculty of Medicine and Health, University Health Care Research Centre, Örebro University, Örebro, Sweden; 2 Centre for Psychiatry Research, Department of Clinical Neuroscience, Karolinska Institutet, Stockholm, Sweden; 3 Stockholm Health Care Services, Stockholm, Sweden; 4 Department of Biological and Medical Psychology, University of Bergen, Bergen, Norway; 5 Department of Clinical Sciences, Division of Adult Psychiatry Faculty of Medicine, Lund University, Lund, Sweden; 6 Department of Medical Epidemiology and Biostatistics, Karolinska Institutet, Stockholm, Sweden; 7 Institute of Neuroscience and Physiology, The Sahlgrenska Academy at Gothenburg University, Gothenburg, Sweden; 8 Center for Social and Affective Neuroscience, Department of Biomedical and Clinical Sciences, Linköping University, Linköping, Sweden; 9 Department of Psychiatry, Region Östergötland, Linköping, Sweden; 10 Department of Medical Sciences, Psychiatry, Uppsala University, Uppsala, Sweden

## Abstract

**Importance:**

Electroconvulsive therapy (ECT), wherein a generalized epileptic seizure is induced, is a treatment for major depressive disorder (MDD). Currently, it is unclear whether there is an association between seizure length and treatment outcome.

**Objective:**

To explore the association between seizure duration, potential confounding variables, and ECT treatment outcome.

**Design, Setting, and Participants:**

This population-based cohort study obtained data from the Swedish National Quality Register for ECT. Patients treated for unipolar MDD with unilateral electrode placement between January 1, 2012, and December 31, 2019, were included. The electroencephalographic (EEG) seizure duration from the first ECT treatment session for each patient was used for analysis. Data analyses were performed between March 2021 and May 2024.

**Main Outcomes and Measures:**

The primary outcome was remission, defined as a cutoff score of less than 10 points on the self-assessment version of the Montgomery-Åsberg Depression Rating Scale within 1 week after ECT. Multivariate logistic regression analysis was performed to calculate odds ratios (ORs) between different seizure duration groups. Furthermore, the associations between concomitant use of pharmacological treatments, seizure duration, and remission rate were explored.

**Results:**

Among the 6998 patients included, 4229 (60.4%) were female and the mean (SD) age was 55.2 (18.6) years. Overall, 2749 patients (39.3%) achieved remission after ECT. Patients with EEG seizure duration of 60 to 69 seconds had the highest remission rates compared with patients with seizure duration of less than 20 seconds (OR, 2.17; 95% CI, 1.63-2.88; *P* < .001). Anticonvulsant medications were associated with shorter seizure duration (eg, lamotrigine: β coefficient [SE], −6.02 [1.08]; *P* < .001) and lower remission rates (eg, lamotrigine: adjusted OR, 0.67; 95% CI, 0.53-0.84; *P* < .001).

**Conclusions and Relevance:**

This study found an association between seizure length and remission from MDD. Use of anticonvulsant medication during ECT was associated with shorter seizure duration and lower remission rates after ECT.

## Key Points

**Question:**

What is the association between seizure duration during electroconvulsive therapy and remission rates from major depressive disorder?

**Findings:**

In this cohort study of 6998 patients, the group with the shortest seizure duration of less than 20 seconds had the lowest remission rate of 27.2% compared with a 39.3% overall remission rate. The highest remission rate was found in the group with seizure duration of 60 to 69 seconds.

**Meaning:**

The study found that seizure duration was associated with electroconvulsive therapy outcome.

## Teaser

This cohort study evaluates the association between the length of electroconvulsive therapy–induced seizure and remission rate of depression in patients with major depressive disorder.

## Introduction

Electroconvulsive therapy (ECT) is a vital treatment for individuals with the most severe depression,^1^ but treatment results differ substantially between patients.^2,3^ One suggested area of research to understand these differences is the interplay among concurrent medication, seizure duration, and treatment effectiveness.

Swedish clinical guidelines currently recommend a motor activity duration of at least 20 seconds, with electroencephalographic (EEG) activity typically exceeding the motoric seizure by 10 to 20 seconds. Seizure duration shorter than this recommendation is considered inadequate and indicates the need for restimulation.^4,5^ Epileptic activity exceeding 120 to 180 seconds is associated with an increased risk of adverse events.^5^ Within this window of 20 to 180 seconds, however, it remains ambiguous whether longer seizure duration is beneficial for therapeutic outcomes. The association between seizure duration and the antidepressant properties of ECT remains uncertain due to limitations of prior studies, such as small sample sizes, inconsistent findings, and a lack of consideration for potential confounding variables.^6,7,8,9^ Larger studies in this area are required, and these studies should take potential confounding factors into account. These confounders include older age,^10,11,12^ diagnostic subgroup,^11,13^ duration of antidepressant medication prior to ECT,^11,12,14^ and concurrent pharmacological treatments with benzodiazepine or lamotrigine.^11,15,16,17^

Thus, the aim of the current large-scale study was to explore the association between seizure duration, potential confounding variables, and ECT treatment outcome using national databases. We hypothesized that longer seizure duration is associated with better treatment outcomes, while anticonvulsive medication is associated with shorter seizures and lower remission rates.

## Methods

### Study Design and Setting

This nationwide register-based, population-based cohort study used the Swedish National Quality Register for ECT (Q-ECT) as the data source. The Q-ECT, established in 2008, collects information on ECT treatments. Since 2012, all hospitals offering ECT in Sweden have been reporting data to Q-ECT. In 2019, the register had a coverage rate of 93% of all patients receiving ECT in Sweden.^18^ The Swedish Ethical Review Authority approved this study and waived the informed consent requirement because all data used were pseudonymized, and individuals were not identifiable at any time. However, patients have accepted being included in the Q-ECT and have been informed that it is used for research. We followed the Strengthening the Reporting of Observational Studies in Epidemiology (STROBE) reporting guideline.

### ECT Method

The treatments were performed in hospitals offering ECT in Sweden. The anesthetic agents used were propofol, 1 to 1.5 mg/kg, or thiopental, 2 to 4 mg/kg. The muscle relaxant used was succinylcholine, 0.5 to 1 mg/kg. The ECT devices used were either Thymatron (Somatics LLC) or MECTA (Mecta Corp). Pulse amplitude, frequency, duration, and charge were individualized for optimal treatment results during the series and initially established mainly based on sex and age but not a multiple of the lowest stimulus that induced some seizure activity.

Seizures were monitored routinely during every treatment, including the observed duration of motor activity, the cardiovascular response, and EEG seizure duration measured with bitemporal EEG electrodes creating a single EEG channel. The EEG seizure duration was selected as the variable for measuring seizure length because it is considered to provide a more accurate representation of the seizure in the brain than the observed motoric seizure and is therefore the variable used in Q-ECT. The analysis included only the seizure duration from the first treatment session because duration data from subsequent sessions were not available.

### Participants and Variables

The inclusion criteria were as follows: unipolar major depressive disorder (MDD) as the indication for treatment according to *International Statistical Classification of Diseases and Related Health Problems, Tenth Revision* codes (F32.1, F32.2, F32.3, F33.1, and F33.2 or F33.3); documented EEG seizure duration from the first treatment session; and evaluation using the self-assessment version of the Montgomery-Åsberg Depression Rating Scale (MADRS-S) within 1 week after the last treatment session. The analysis included patients treated between January 1, 2012, and December 31, 2019, because Q-ECT became national in 2012. In cases where a patient received multiple ECT series during this period, only the first registered treatment series was analyzed. Furthermore, only those who underwent treatment with unilateral electrode placement were included.

To assess the outcome, the MADRS-S was used within 1 week after the last ECT session. The MADRS-S consists of 9 items, with the patient rating each item from 0 (no distress at all) to 6 (maximum distress).^19^ The maximum score possible was 54 points, and a cutoff score of less than 10 points was defined as remission in this study.^20^

### Statistical Analysis

Data were managed and analyzed using SPSS Statistics 25.0 (IBM Corp). Seizure duration was categorized to assess nonlinear associations, and the characteristics of the study population were stratified into categories. Anesthetic dose and electrical charge were divided into 2 groups based on standardized median values for age and sex categories (eTable 1 in Supplement 1). Logistic regression models were created with seizure duration as an independent variable to calculate odds ratios (ORs) for remission. Age and subtype of MDD (with or without psychotic features) were adjusted for in the main model. A forest plot was created to demonstrate the association. In addition, electric charge, anesthetic agent, and dose were adjusted for because they are potential confounding variables in a sensitivity model. Linear regression analyses were performed to compare the means in seizure duration among patients with or without concurrent pharmacological treatments. The null hypothesis was rejected at *P* < .05. Nagelkerke *R*^2^ was used to estimate the goodness of fit in logistic regression models and adjusted *R*^2^ in linear regression models. Two-sided *P* < .05 indicated statistical significance. Data analyses were performed between March 2021 and May 2024.

## Results

### Descriptive Data

Among the 6998 patients who met the inclusion criteria, 4229 (60.4%) were female and 2769 (39.6%) were male, with a mean (SD) age of 55.2 (18.6) years. Further details on the patient population are presented in Table 1. The number of patients in each seizure category are provided in the eFigure in Supplement 1.

**Table 1.** Characteristics of the 6998 Study Patients

Characteristic	Patients, No. (%)
Sex	
Female	4229 (60.4)
Male	2769 (39.6)
Age categories, y	
<30	848 (12.1)
30-49	1870 (26.7)
50-70	2513 (35.9)
>70	1767 (25.3)
Diagnosis	
MDD without psychotic features	5646 (80.7)
MDD with psychotic features	1352 (19.3)
Concurrent pharmacological treatment	
Antidepressants	6232 (89.1)
Lamotrigine	471 (6.7)
Benzodiazepines	3335 (47.7)
Lithium	649 (9.3)
Antipsychotics	259 (46.6)
Other anticonvulsants	480 (6.9)
Anesthetic agent	
Propofol	2343 (33.5)
Thiopental	4312 (61.6)

Abbreviation: MDD, major depressive disorder.

### Main Results

Among the included patients, 2749 (39.3%) achieved remission after treatment. The group with the shortest seizure duration (<20 seconds) during the first treatment session had the lowest remission rate of 27.2%. In univariate analysis, the group with a seizure duration of 60 to 69 seconds had the highest remission rate compared with the group with the shortest seizure duration (OR, 2.17; 95% CI, 1.63-2.88; *P* < .001). Both univariate and multivariate regression models showed an association between seizure duration and remission rate (Table 2, Figure). In the sensitivity model, after also adjusting for the anesthetic agent, dose, and electrical charge, the association was more pronounced (adjusted OR [AOR], 2.59; 95% CI, 1.93-3.48; *P* < .001). There was also an association between remission and seizure duration as a continuous variable in the sensitivity model (AOR, 1.07; 95% CI, 1.01-1.01; *P* < .001).

**Table 2.** Remission as a Variable Associated With Seizure Duration, Unadjusted and Adjusted for Age and Depression Subtype With Psychotic Features

EEG seizure duration, s	OR (95% CI)^a^	*P* value	AOR (95% CI)**^a^**	*P* value
<20	1 [Reference]	NA	1 [Reference]	NA
20-29	1.38 (1.04-1.83)	.03	1.56 (1.16-2.09)	.003
30-39	1.64 (1.25-2.14)	<.001	2.09 (1.58-2.77)	<.001
40-49	1.70 (1.30-2.22)	<.001	2.23 (1.69–2.95)	<.001
50-59	2.07 (1.58-2.72)	<.001	2.45 (1.85-3.25)	<.001
60-69	2.17 (1.63-2.88)	<.001	2.52 (1.88-3.39)	<.001
70-236	1.87 (1.42-2.46)	<.001	2.45 (1.84-3.28)	<.001

Abbreviations: AOR, adjusted odds ratio; EEG, electroencephalographic; NA, not applicable; OR, odds ratio.

^a^ The model fit according to Nagelkerke *R*^2^ was 0.009 in the univariate model and 0.13 in the multivariate model.

**Figure.**
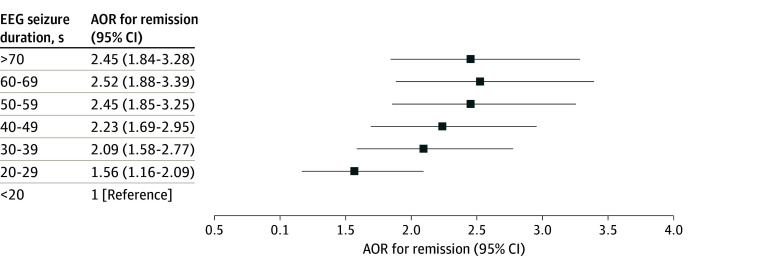
Remission as a Variable Dependent on Electroencephalographic (EEG) Seizure Duration Error bars represent 95% CIs. AOR indicates adjusted odds ratio.

For those who achieved remission after treatment, the median (IQR) number of sessions was 7 (6-9). The corresponding median (IQR) number of sessions for the nonremission group was 8 (6-10). The mean (SD) values of the ECT setting were as follows: pulse width of 0.5 (0.1) milliseconds, frequency of 62 (20) Hz, duration of 6.8 (1.3) seconds, and current of 842 (55) mA. Further details are provided in eTable 2 in Supplement 1.

### Other Analyses

Linear regression analyses revealed that older age was associated with shorter seizure duration (β coefficient [SE], −0.11 [0.02]; *P* < .001). Moreover, older age was associated with higher remission rates (Table 3). The MDD subtype with psychotic features was associated with higher remission rates (OR, 2.39; 95% CI, 2.12-2.69; *P* < .001), but there was no association with seizure duration (β coefficient [SE], 0.39 [0.68]; *P* = .56) (Table 3).

**Table 3.** Association of Age and Depression Subtype With Psychotic Features With Remission and Seizure Duration

Characteristic	Remission rate, OR (95% CI)	*P* value	Seizure duration, β coefficient (SE)	*P* value
Age, y	1.03 (1.03-1.04)	<.001	−0.11 (0.02)	<.001
MDD subtype with psychotic features	2.39 (2.12-2.69)	<.001	0.39 (0.68)	.56

Abbreviations: MDD, major depressive disorder; OR, odds ratio.

Concurrent use of lamotrigine (β coefficient [SE], −6.02 [1.08]; *P* < .001), benzodiazepines (β coefficient [SE], −2.55 [0.54]; *P* < .001), antipsychotics (β coefficient [SE], −1.66 [0.56]; *P* = .003), or other anticonvulsants (β coefficient [SE], −8.43 [1.06]; *P* < .001) was associated with shorter seizure duration. Lamotrigine (AOR, 0.67; 95% CI, 0.53-0.84; *P* < .001), benzodiazepines (AOR, 0.76; 95% CI, 0.69–0.84; *P* < .001), and other anticonvulsants (AOR, 0.53; 95% CI, 0.42-0.66; *P* < .001) were also associated with significantly lower remission rates. A higher electrical charge was associated with significantly shorter seizure duration (β coefficient [SE], −3.32 [0.54]; *P* < .001) and higher remission rates (AOR, 1.15; 95% CI, 1.04-1.28; *P* = .005). Thiopental was associated with longer seizure duration and lower remission rates compared with propofol’s duration (β coefficient [SE], 2.49 [0.57]; *P* < .001) and remission (AOR, 0.87; 95% CI, 0.79-0.97; *P* = .02). Higher anesthetic dose was associated with shorter seizure duration (β coefficient [SE], −0.67 [0.56]; *P* = .23) and lower remission rates (AOR, 0.84; 95% CI, 0.76-0.94; *P* < .001) (Table 4).

**Table 4.** Association of Potential Confounders in Remission With Seizure Duration After Adjustment for Age and Depression Subtype With Psychotic Features

Potential confounder	Remission rate, AOR (95% CI)^a^	*P* value	Seizure duration, β coefficient (SE)	*P* value
Concurrent pharmacological treatments				
Antidepressants	1.13 (0.96-1.33)	.15	1.04 (0.86)	.23
Lamotrigine	0.67 (0.53-0.84)	<.001	−6.02 (1.08)	<.001
Benzodiazepines	0.76 (0.69–0.84)	<.001	−2.55 (0.54)	<.001
Lithium	1.01 (0.85-1.20)	.93	1.12 (0.93)	.23
Antipsychotics	0.92 (0.83-1.02)	.12	−1.66 (0.56)	.003
Other anticonvulsants	0.53 (0.42-0.66)	<.001	−8.43 (1.06)	<.001
Anesthetic agent				
Propofol	1 [Reference]	NA	NA	NA
Thiopental	0.87 (0.79-0.97)	.02	2.49 (0.57)	<.001
Anesthetic dose				
Low dose	1 [Reference]	NA	NA	NA
High dose	0.84 (0.76-0.94)	<.001	−0.67 (0.56)	.23
Electrical charge				
Low dose	1 [Reference]	NA	NA	NA
High dose	1.15 (1.04-1.28)	.005	−3.32 (0.54)	<.001

Abbreviations: AOR, adjusted odds ratio; NA, not applicable.

^a^ Nagelkerke *R*^2^ in the logistic regression models was between 0.12 and 0.13 for all significant models. Adjusted *R*^2^ in the linear models was between 0.01 and 0.02 for all significant models.

## Discussion

The findings of this study support the hypothesis of an association between seizure duration during the first treatment session and remission within 1 week after ECT. This result is consistent with data from the double-blind experimental study by Cronholm and Ottosson.^21^ In their study, the group of patients whose seizures were shortened by lidocaine medication had significantly worse treatment outcomes compared with the control group, although the lidocaine-modified group had a higher number of total treatment sessions.^21^ Similarly, a larger register-based study by Kronsell and colleagues^22^ found that patients receiving low-dose anesthetics had superior treatment outcomes compared with those receiving high-dose anesthetics. The 2 patient groups differed in seizure duration and number of treatment sessions, with the low-dose group having longer seizure duration, requiring shorter treatment series, and having higher remission rates.^22^ Previous observational studies, which did not find an association between seizure duration and treatment response, had smaller sample sizes (29 patients^23^ and 40 patients,^23^ respectively).

This study also showed that older age was associated with better treatment outcomes, which aligns with several previous findings.^10,11,12^ When adjusting for age in a multivariate regression model, the association between remission and seizure duration was more pronounced since older age was associated with shorter seizure duration and therefore could have been a confounder in previous research.

Concurrent medication with lamotrigine or benzodiazepines was found to be associated with shorter seizure duration, with the association being most pronounced for lamotrigine treatment. Furthermore, patients treated with lamotrigine had significantly lower remission rates (Table 4). These findings align with those of previous research in the field and suggest that the reduced seizure duration associated with these medications may be a factor in the lower therapeutic outcome of ECT.^11,15,16,17^ It may seem surprising that medications with anticonvulsant properties are combined with ECT given that a high-quality seizure is important for the optimal ECT outcome. The lack of data associating seizure duration or anticonvulsant medication with therapeutic outcomes could be an explanation, along with the frequent use of these medications for MDD that did not respond to antidepressants. The concurrent use of anticonvulsants or benzodiazepines in some hospitals may partly explain the marked difference in the treatment effect of ECT reported in some recent studies.^2,3,24^ Yet, the association between lower remission rates and lamotrigine or benzodiazepine treatment could be explained by factors other than seizure duration. The severity of the underlying condition along with the potential comorbidities leading to the prescription of these medications may themselves be confounding factors in the lower remission rate after ECT among patients receiving lamotrigine or benzodiazepines compared with those not receiving these medications.

Higher electrical charges were found to be associated with higher remission rates and shorter seizure duration, which is consistent with previous research.^25,26^ The association between thiopental vs propofol and longer seizure duration was also expected.^27^ Nevertheless, the association of higher electrical dose with shorter seizure duration and higher remission rates, and thiopental with longer seizure duration and lower remission rates, highlight the underlying complexity of these associations and call for caution in interpreting the results.

### Strengths and Limitations

The strengths of this study are the combination of a large study cohort, a nationwide register-based design, and use of a standardized assessment tool for remission rates, which not only provided the statistical power to adjust for potential confounding variables but also facilitated the generalization of the results. The main model associating seizure duration with remission indicated age and MDD with psychotic features as important potential confounding factors but did not control for anesthetic agent or dose, electrical charge, or concurrent medication use because these factors may affect both seizure duration and remission rate, thus being intermediate in the potential causal chain. However, they were included in a sensitivity model, which showed similar associations.

The limitations of this study include the uncertainty of whether the observed association between seizure length and treatment outcome is causal. We were not able to control for the possible confounder of duration of antidepressant treatment. Furthermore, with the mechanics of seizures being complex, the seizure duration may be associated with good treatment quality, but other factors, such as seizure intensity or seizure generalization, might play a larger role in the antidepressant properties of ECT. These aspects of the seizure were not investigated in this study. Additionally, this study investigated only patients treated with unilateral electrode placement because this is the most common electrode placement in Sweden. Therefore, the results need to be confirmed in samples treated by bitemporal electrode placement before the conclusions can be generalized to treatment with bitemporal electrode placement.

## Conclusions

To our knowledge, this cohort study is the largest yet supporting the association between seizure length and remission from MDD after ECT. Seizure duration appeared to be indicative of adequate treatment quality. The use of anticonvulsant medication during ECT was associated with shorter seizure duration and lower remission rates.

## Notes

Supplement 1. **eTable 1.** The Medians of Electrical Charge and Anesthetic Dosage of Patients in Different Age and Sex Categories **eTable 2.** Number of Treatment Sessions Among Patients Within Different Seizure Duration Categories, and With and Without Remission **eFigure.** Number of Patients in Each Seizure Duration Group

Supplement 2.
Data Sharing Statement
