# Supplementary material for: Seizure Duration and Electroconvulsive Therapy in Major Depressive Disorder
Source: JAMA Netw Open. 2024 Jul 25;7(7):e2422738. doi: 10.1001/jamanetworkopen.2024.22738 (PMC11273235; doi:10.1001/jamanetworkopen.2024.22738)

Supplemental Online Content

Gillving C, Ekman CJ, Hammar Å, et al. Seizure duration and electroconvulsive therapy in major depressive disorder. *JAMA Netw Open*. 2024;7(7):e2422738. doi:10.1001/jamanetworkopen.2024.22738

eTable 1. The Medians of Electrical Charge and Anesthetic Dosage of Patients in Different Age and Sex Categories

eTable 2. Number of Treatment Sessions Among Patients Within Different Seizure Duration Categories, and With and Without Remission

eFigure. Number of Patients in Each Seizure Duration Group

This supplemental material has been provided by the authors to give readers additional information about their work.

© 2024 Gillving C et al. *JAMA Network Open*

eTable 1. The medians of electrical charge and anesthetic dosage of patients in different age and sex categories.

Category	Electrical charge (mC)	Anesthetic dose (mg)	
		Propofol	Thiopental
**Female**			
0-29	203	100	250
30-49	288	100	250
50-69	352	80	250
≥70	400	60	200
**Male**			
0-29	240	120	325
30-49	288	120	300
50-69	384	100	300
≥70	432	80	225

© 2024 Gillving C et al. *JAMA Network Open*

eTable 2. Number of treatment sessions among patients within different seizure duration categories, and with and without remission.

Category	Median	Interquartile range
**Seizure duration (seconds)**		
<20	8	6-10
20-29	8	6-10
30-39	8	6-9
40-49	7	6-9
50-59	7	6-9
60-69	7	6-9
≥70	7	6-9
**Remission**		
Yes	7	6-9
No	8	6-10

© 2024 Gillving C et al. *JAMA Network Open*

eFigure. Number of patients in each seizure duration group

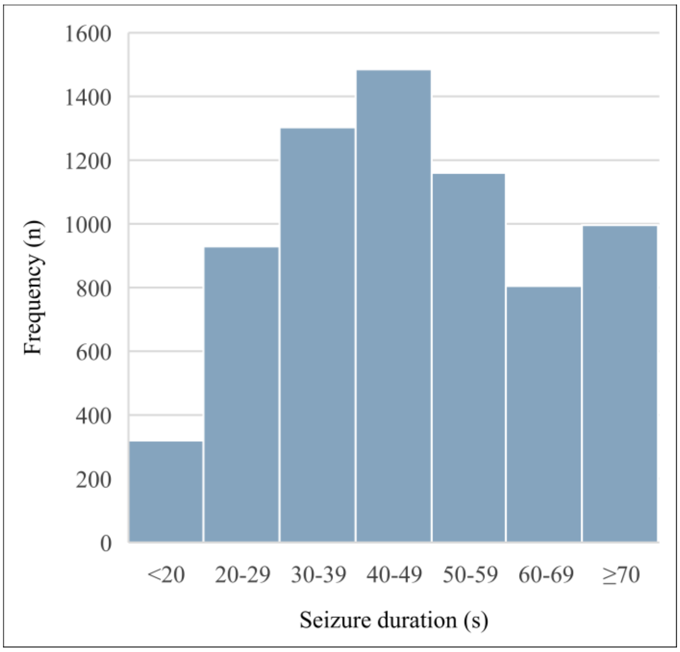

Supplement: Supplement 1. — eTable 1. The Medians of Electrical Charge and Anesthetic Dosage of Patients in Different Age and Sex Categories eTable 2. Number of Treatment Sessions Among Patients Within Different Seizure Duration Categories, and With and Without Remission eFigure. Number of Patients in Each Seizure Duration Group [file jamanetwopen-e2422738-s001.pdf]
