# Supplementary material for: Seizure Duration and Electroconvulsive Therapy in Major Depressive Disorder
Source: JAMA Netw Open. 2024 Jul 25;7(7):e2422738. doi: 10.1001/jamanetworkopen.2024.22738 (PMC11273235; doi:10.1001/jamanetworkopen.2024.22738)
Supplement: Supplement 2. — Data Sharing Statement [file jamanetwopen-e2422738-s002.pdf]

Data Sharing Statement

Gillving. Seizure Duration and Electroconvulsive Therapy in Major Depressive Disorder. *JAMA Netw Open*. Published July 25, 2024. doi:10.1001/jamanetworkopen.2024.22738

Data
Data available: No

Additional Information
Explanation for why data not available: Data will be made available from the corresponding author upon reasonable request.
